# Peer Review of “Telerehabilitation for People With Physical Disabilities and Movement Impairment: A Survey of United Kingdom Practitioners”

**DOI:** 10.2196/35850

**Published:** 2022-01-03

**Keywords:** telerehabilitation, physical disabilities, movement impairment, remote assessments, telehealth, rehabilitation, training, health care practitioners, physiotherapy, occupational therapy

*This is a peer-review report submitted for the paper “Telerehabilitation for People With Physical Disabilities and Movement Impairment: A Survey of United Kingdom Practitioners.”*

## Round 1 Review

### General Comments

This paper [1] reports a mixed methods survey of UK practitioners’ use of telerehabilitation for people with physical disabilities and movement impairment. It investigated practitioners’ experiences of telerehabilitation (including use, perceived benefits and obstacles, and physical outcomes assessed remotely), perceived confidence and competence, knowledge and training needs, and best practice and recommendations. It provides practical clinical recommendations for practitioners delivering telerehabilitation and has identified a number of training needs. This is very important research due to the huge uptake of virtual consultations/remote rehabilitation due to the COVID-19 pandemic and much uncertainty over its effectiveness and best practice. This paper is well written and clear to understand. I have a couple of minor comments.

#### Minor Comments

1. The Data Analysis section could include more detail regarding the qualitative analysis method used. The authors state they followed the guidance of Braun and Clarke but more detail on exactly how this was conducted would be beneficial to the readers. Relatedly, it is unclear which results they used this method for; it appears it is the *concerns of practitioners regarding the reliability and validity of remote physical assessments* (Table 4) and *practitioners’ perceived benefits and obstacles of video-based consultations* (Figure 2) sections, but this is unclear. Perhaps the authors could clarify exactly how they conducted their qualitative analysis and which data/results they used this method for.

2. There are a number of clinical practice implications from the results of this study, particularly with the recommendations for carrying out telerehabilitation in Textbox 1. It would be useful to have a clinical implications section in the Discussion, outlining how the results of this study might be useful for clinical practice.
